# Characterisation of South African field *Ehrlichia ruminantium* using multilocus sequence typing

**DOI:** 10.4102/ojvr.v90i1.2119

**Published:** 2023-11-14

**Authors:** Zinathi Dlamkile, Luis Neves, Darshana Morar-Leather, Christopher Brandt, Alri Pretorius, Helena Steyn, Junita Liebenberg

**Affiliations:** 1 Department of Veterinary Tropical Diseases, Faculty of Veterinary Science, University of Pretoria, Pretoria, South Africa; 2 Centro de Biotecnologia-UEM, Eduardo Mondlane University, Maputo, Mozambique; 3 Department of Vaccines and Diagnostics Development, Onderstepoort Veterinary Research Institute, Agricultural Research Council, Pretoria, South Africa

**Keywords:** *Ehrlichia ruminantium*, heartwater, characterisation, pCS20, multilocus sequence typing, MLST, phylogenetic, PCR

## Abstract

Heartwater, one of the major tick-borne diseases of some domestic and wild ruminants in Africa, is caused by *Ehrlichia ruminantium*. The genetic diversity of *E. ruminantium* isolates renders the available vaccine ineffective against certain virulent isolates. To better understand the *E. ruminantium* genotypes in South Africa, a total of 1004 *Amblyomma hebraeum* tick deoxyribonucleic acid (DNA) samples from cattle in three South African provinces were tested by pCS20 Sol1 real-time polymerase chain reaction (qPCR) and characterised by multilocus sequence typing (MLST) using five housekeeping genes. Out of 1004 samples tested, 222 (22%) were positive for *E. ruminantium*. The occurrence of *E. ruminantium* in Mpumalanga, KwaZulu-Natal and Limpopo provinces was 19%, 22% and 27%, respectively. The *E. ruminantium* positive samples were screened for housekeeping genes and sequenced. Phylogenetic analysis revealed three main lineages: clade 1 made up of worldwide isolates (eastern, southern Africa, and Caribbean isolates), clade 2 comprised only West African isolates and clade 3 consisted of Omatjenne, Kümm2 and Riverside. Some study sample sequences were not identical to any of the reference isolates. However, they could all be grouped into the worldwide clade. Genetic variation in the sequenced regions was observed in the form of single nucleotide polymorphisms (SNPs). Using MLST to characterise *E. ruminantium* field isolates allowed the South African genotypes to be clearly distinguished from the distinct West African isolates.

**Contribution:**

Characterisation of *E. ruminantium* field isolates is important for the control of heartwater and contributes to preliminary knowledge required for the development of a more practical vaccine against heartwater.

## Introduction

*Ehrlichia ruminantium*, previously known as *Cowdria ruminantium*, is the causative agent of heartwater in some wild and domestic ruminants (Bezuidenhout 2009). Heartwater occurs in regions where ticks from the *Amblyomma* genus (*E. ruminantium* vectors) are present (Petney, Horak & Rechav 1987). In South Africa, *E. ruminantium* is transmitted by *Amblyomma hebraeum* ticks, while in other countries in sub-Saharan Africa, Indian Ocean islands and the Caribbean, it is predominantly transmitted by *Amblyomma variegatum* ticks (Walker & Olwage 1987). Heartwater is endemic in six of the nine provinces of South Africa: Limpopo (LP), Mpumalanga (MP), KwaZulu-Natal (KZN), Gauteng (G), Eastern Cape (EC) and North West province (NW) (Purnell 1984). Livestock farmers who are interested in introducing high-producing susceptible exotic breeds to upgrade local stock are hindered by heartwater as mortality rate of susceptible animals ranges from 20% to 90% (Mahan et al. 1992).

Currently there is no efficient vaccine against heartwater. The commercially available vaccine in South Africa consists of sheep blood infected with the Ball3 isolate and employs the ‘infection and treatment’ vaccination method (Van der Merwe 1987). However, the Ball3 vaccine does not cross-protect against some virulent isolates. The limited cross-protection is caused by high genetic diversity of *E. ruminantium* (Cangi et al. 2016). A prerequisite to developing effective control strategies for heartwater is to understand the genetic diversity of *E. ruminantium* isolates. Different methods of genotyping *E. ruminantium* have been investigated and are mostly based on conserved molecular markers. Allsopp et al. (2003) used the genes *groESL*, citrate synthase (*gltA*), 16S rRNA and pCS20 (a cloned deoxyribonucleic acid [DNA] fragment containing two partial open reading frames) to phylogenetically characterise *E. ruminantium* isolates. The sequences of *groESL, gltA* and pCS20 revealed single nucleotide polymorphisms (SNPs) spreading throughout the sequenced regions (Allsopp et al. 2003). In addition to the aforementioned markers, Allsopp and Allsopp (2007) used the functional genes, *rnc, ctaG, ftsZ, nuoB* and *sodB*, to characterise *E. ruminantium* isolates from different geographic origins. Isolates from Southern and East Africa were found to group together while isolates from West Africa and one from Southern Africa were observed to group together, revealing a great genetic variability between Southern and East African *E. ruminantium* isolates.

Multilocus sequence typing (MLST) is a technique that is widely used for molecular characterisation of bacteria (Maiden 2006). Adakal et al. (2009) developed an MLST scheme for *E. ruminantium* based on eight housekeeping genes (*gltA, groEL, lepA, lipA, lipB*, secY, *sodB* and *sucA*). The MLST was used by Nakao et al. (2011) on a panel of reference isolates and field samples from geographically diverse origins. In both studies, *sodB* was the most conserved locus among the isolates examined. Conversely, the locus with the highest percentage of polymorphic sites was *secY*. The MLST allowed for closely related isolates to be distinguished, with high degree of genetic heterogeneity observed among them. *E. ruminantium* isolates cluster into two main groups: Group 1 (West Africa) and Group 2 (worldwide), which are represented by West, East and Southern Africa, Indian Ocean and Caribbean isolates when using MLST (Cangi et al. 2016; Nakao et al. 2011). The aim of the current study is to genetically characterise *E. ruminantium* field isolates currently circulating in three South African provinces using MLST.

## Materials and methods

### Study areas

**FIGURE 1:**
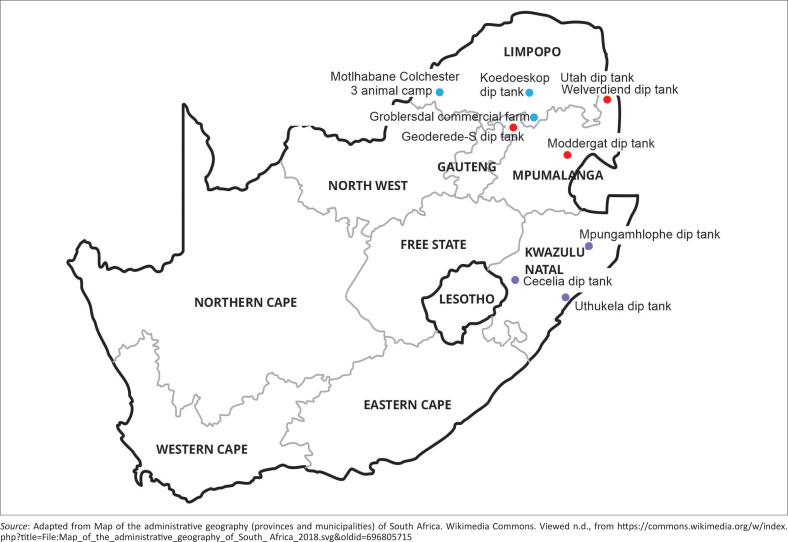
Map of South Africa showing where sample collection areas (coloured dots) are situated in the provinces of Limpopo, Mpumalanga and KwaZulu-Natal.

Sample collection areas are shown in Figure 1. For MP samples, DNA extracted from ticks collected in Utah and Welverdiend dip tanks was obtained by Mazhetese et al. (2022). Ticks were collected from cattle in Moddergat and Geoderede-S dip tanks in MP. In LP, ticks were collected from cattle in Koedoeskop dip tank, Motlhabane Colchester 3 animal camp and a commercial farm in Groblersdal. In KZN, ticks were collected from cattle at the following dip tanks: Cecelia, Uthukela and Mpungamhlophe.

### Tick collection

Tick sampling was carried out between October 2020 and January 2022 where rainfall was expected to increase and tick population to have been abundant. *Amblyomma hebraeum* ticks were handpicked from cattle with permission from the cattle owners at dip tanks. About 3–5 adult ticks were handpicked from each animal and the minimum number of cattle sampled per collection site was 20. Collected ticks were kept alive in a humid environment and transported to the Research and Training laboratories, Department of Veterinary Tropical Disease, University of Pretoria adhering to the biosafety rules and regulation stipulated by the Section 20 permit (DALRRD). Using a stereo microscope, the ticks were identified based on entomological keys by Walker et al. (2003). Microscopy is done to correctly identify *A. hebraeum* ticks to be tested.

### Deoxyribonucleic acid extraction

Individual ticks were washed with 70% ethanol, rinsed with distilled water and air-dried in a laminar flow. For each tick, DNA was extracted from two legs that were cut close enough to the body of the tick to include the hemolymph. The DNA was extracted using the Qiagen DNeasy Blood and Tissue kit (Qiagen, Germany) according to the manufacturer’s instructions. The tick legs were cut into small pieces in 180 µL lysis buffer ATL. Proteinase K digestion was carried out at 56 °C overnight. Genomic DNA was eluted from the column with 100 µL elution buffer. The DNA was kept at −20 °C until further analysis was conducted.

### Detection of *Ehrlichia ruminantium* using pCS20 real-time polymerase chain reaction

Detection of *E. ruminantium* was performed by targeting a conserved genomic DNA fragment known as the pCS20 region using the TaqMan real-time polymerase chain reaction (qPCR), previously described by Cangi et al. (2017). Each reaction was performed in a final volume of 25 µL containing 2 µL DNA as template, a final concentration of 0.25 µM of each primer (pCS20 Sol1 Forward and pCS20 Sol1 Reverse in Table 1), 0.2 µM probe (Sol1 TM probe in Table 1) and TaqMan Universal PCR MasterMix 1X (Thermo Fischer Scientific, England). Thermal cycling consisted of one cycle of uracil-N-glycosylase (UNG) incubation at 50 °C for 2 min and one cycle of AmpliTaq Gold pre-activation at 95 °C for 10 min. This was then followed by 40 cycles of denaturing at 95 °C for 15 s and annealing at 55 °C for 1 min and kept at 4 °C.

**TABLE 1:** Oligonucleotides used for pCS20 qPCR and polymerase chain reaction amplification of housekeeping genes.

Primers/probe	Sequence 5′-3′	Annealing temperature (°C)	Product size (bp)
pCS20 Sol1F	ACAAATCTGGYCCAGATCAC	55	280
pCS20 Sol1 R	CAGCTTTCTGTTCAGCTAGT	-	-
Sol1 TM probe	6-FAM-ATCAATTCACATGAAACATTACATGCAACTGG-BHQ1	-	-
*lipA* F	GGATCCTCATGAGCCTCAAA	62	430
*lipA* R	CTGCCGACCTTAAATCATCCATA	-	-
*lipB* F	TGGAAGATTGAATCCTTACCTG	59	452
*lipB* R	CCATGATATGTTATCCATTTTC	-	-
*secY* F	CCAGGCATTAATCCAGATGT	61	690
*secY* R	GCGGAACATATGTAGATGCAGT	-	-
*sodB* F	TGCCAGAACTGCCTTATCAA	61	507
*sodB* R	AAGCGTGTTCCCATACATCC	-	-
*sucA* F	TGAAAGGCTTTGGCTACAGG	59	566
*sucA* R	CCTGACCAATTACAGCAGCA	-	-

bp, base pairs.

### Characterisation of *Ehrlichia ruminantium* isolates using multilocus sequence typing

Multilocus sequence typing was conducted on pCS20 qPCR positive samples using the housekeeping genes *lipA, lipB, secY, sodB* and *sucA* to characterise *E. ruminantium* field samples (Cangi et al. 2016). Each housekeeping gene was amplified using conventional PCR with the primers listed in Table 1. Polymerase chain reaction amplification was performed in a 20 µL reaction with 1X Phusion Flash High Fidelity PCR Master Mix (Thermo Fisher Scientific, Lithuania), 2.5 µL template DNA and 0.5 µM of each primer (each primer set with its annealing temperature is indicated in Table 1). Polymerase chain reaction cycling conditions consisted of 94 °C for 3 min; followed by 40 cycles of 94 °C for 50 s, primer annealing (see temperature in Table 1) for 50 s and extension at 72 °C for 50 s, followed by a final extension of 72 °C for 10 min. The PCR products were run on a 1.5% agarose gel stained with ethidium bromide and visualised with ultraviolet (UV) light illumination and photography. The PCR products were sequenced at Inqaba Biotec using Sanger sequencing.

### Sequences and phylogenetic analysis

Sequences obtained from the housekeeping genes were processed using CLC Genomics Workbench version 7.5.1 (CLC Bio, Boston, MA, United States [US]). The homologous sequences of the five genes were identified in the genome sequences available from NCBI (https://www.ncbi.nlm.nih.gov/genome/microbes/): Welgevonden (NC_005295.2/ CR767821.1), Gardel (NC_006831.1/CR925677.1), Blaauwkrans (CP063043), Grootvallei (CP040120), Kwanyanga (CP040119), Mara87/7 (CP040118), Nonile (CP040117), Springbokfontein1 (CP040116), Springbokfontein2 (CP040115), Springbokfontein4 (CP040114), Springbokfontein5 (CP040113), Springbokfontein6 (CP040112), Springbokfontein7 (CP040111), Um Banein (CP063044), Crystal Springs (BDDK01000001 to BDDK01000034), Senegal (NZ_MQUJ00000000.1), Sankat 430 (BDDN01000001 to BDDN01000183), Kümm2 (CP033456), Omatjenne (CP033455) and Riverside (CP033454), as well as the incomplete genome sequence of Ball3 (personal data, property of Dr Junita Liebenberg).

Alignments of sequences from the housekeeping genes were constructed using the Multiple Alignment using Fast Fourier Transform (MAFFT) (version 7) programme (Katoh & Standley 2013) and manually edited using BioEdit (version 7.2.5). A phylogenetic tree was constructed using concatenated nucleotide sequences of the housekeeping genes, *sodB, secY, lipB* and *lipA* partial sequences, using the maximum likelihood (ML) method in MEGA7. The reliability of the internal branches was assessed using bootstrapping (100 bootstrap replicates). Graphical representation and editing of the phylogenetic trees were performed with MEGA7 and Paint Tool for Windows 10.0.

### Statistical analysis

Distributional patterns for the occurrence of *E. ruminantium* were described for each province and for study areas within each province. We determined how provinces and study areas differed in their occurrence using the non-parametric Kruskal–Wallis test. The provinces and study areas were the independent variables while the occurrence served as the dependent variable. To measure the difference between the various provinces and study areas within the provinces, a post hoc test (Tukey’s honestly significant difference) was performed at a significance level of 5% (*p* < 0.05). All statistical analyses were performed in R Console version 3.2.1.

### Ethical considerations

The study was approved by the institutional research ethics committee, and the part of the study involving animals was performed in accordance with the stipulation of the Animal Ethics Committee at the University of Pretoria, Faculty of Veterinary Science (research and animal ethical clearance number: REC205-19). Permission to conduct the study in terms of Section 20 of the *Animal Diseases Act 1984* (Act No. 35 of 1984) was granted by the Department of Agriculture, Land Reform and Rural Development (DALRRD).

## Results

The collected ticks were verified to be *A. hebraeum*. A total of 1004 DNA samples were extracted from *A. hebraeum* ticks collected from cattle in MP, LP and KZN provinces of South Africa and were tested for *E. ruminantium*.

### Detection and prevalence of *Ehrlichia ruminantium* by pCS20 qPCR

The number of ticks collected in each study area and that of pCS20 positive ticks is shown in Appendix 1, Table 1-A1. Occurrence of *E. ruminantium* was 19%, 22% and 27% in ticks collected in MP, KZN and LP provinces, respectively. The overall *E. ruminantium* infection rate in ticks collected in the three *E. ruminantium* endemic provinces of South Africa was 22%. The positivity rate was the highest in samples collected in Moddergat dip tank followed by Motlhabane Colchester animal camp (43% and 34%, respectively).

**FIGURE 2:**
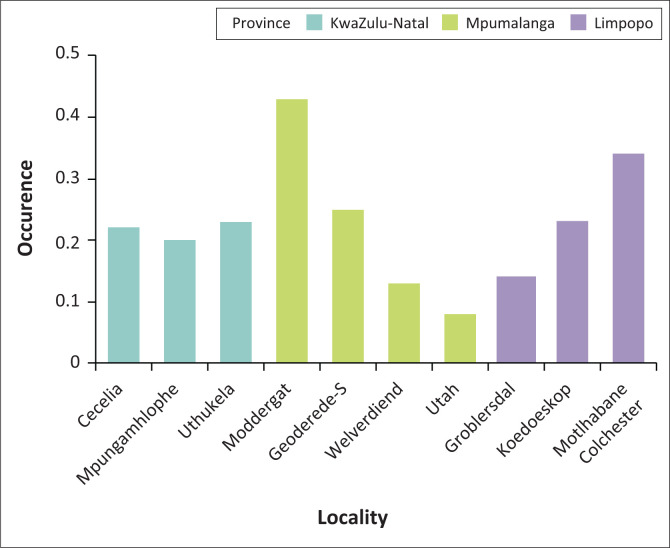
Bar graph showing the occurrence of *Ehrlichia ruminantium* in *Amblyomma hebraeum* ticks collected from cattle in the study areas.

Comparing all the provinces together, the Kruskal–Wallis test showed that even though MP had the highest occurrence, this was not significant (*p* > 0.0946). Also, the post hoc test across the provinces showed no significant difference (Figure 2). Pairwise comparison across the study areas showed significant differences between all the study areas in MP (*p* < 0.005), study areas within LP province (*p* < 0.005), but no significant differences between the study areas in the KZN province (*p* > 0.005; Figure 2).

### Characterisation of *Ehrlichia ruminantium* isolates using multilocus sequence typing

All 222 pCS20-positive samples were tested for the five housekeeping genes (*lipA, lipB, secY, sodB* and *sucA*) to characterise *E. ruminantium* field isolates. For some samples, amplification was not successful for all the MLST genes, which limited the possibility of obtaining sequences for all loci for all samples. A total of 524 amplicons (all loci) were sent for sequencing. Out of the 28 samples from Utah and Welverdiend dip tanks, 14 allowed for the successful amplification of all the housekeeping genes, with *sodB* amplicons available in 27 samples (results not shown).

All the amplicons of samples for which the housekeeping genes were amplified were sequenced (Inqaba Biotechnical Industries [Pty] Ltd) and found to be 99.4% – 100% identical to selected reference isolates: Ball3, Nonile, Kwanyanga, Mara87-7, Grootvallei, Welgevonden and Springbokfontein 1; 2; 4; 5; 6; 7 for each gene (http://blast.ncbi.nlm.nih.gov/Blast.cgi). Because the identity of some samples was the same, a small number of sequences was included in the construction of the phylogenetic tree.

**FIGURE 3:**
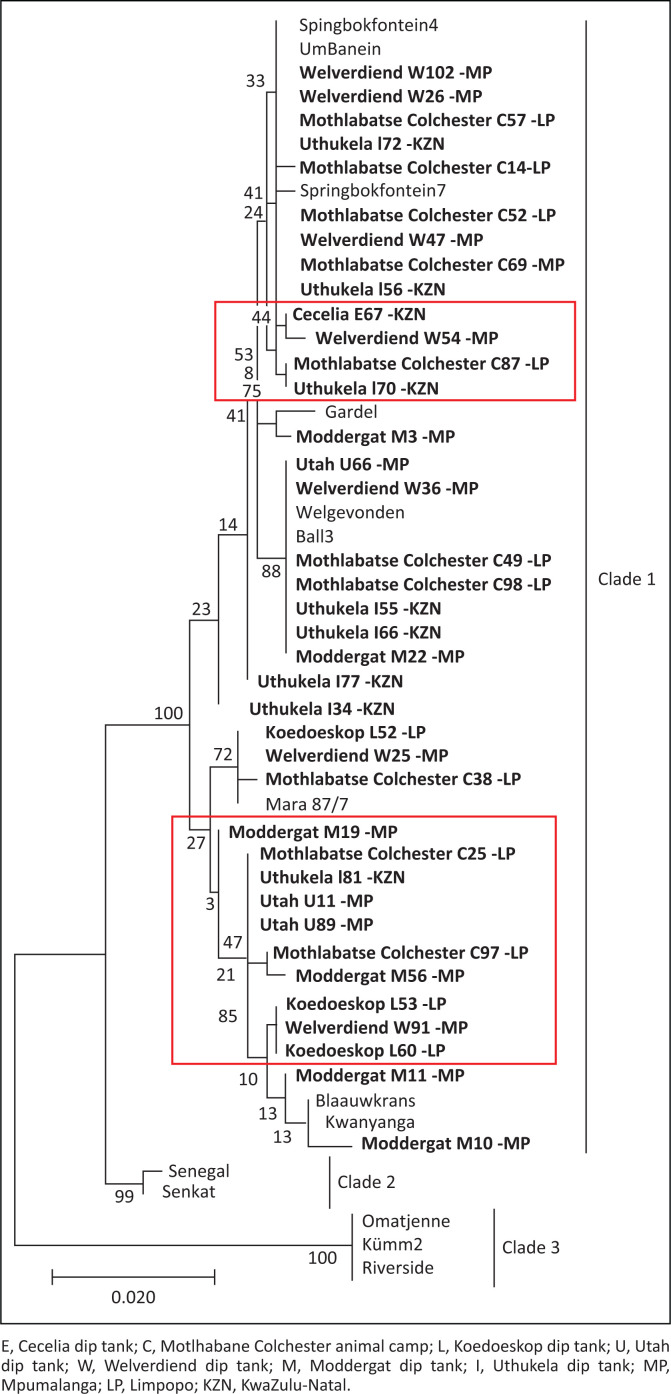
Phylogenetic tree showing relationship between *Ehrlichia ruminantium* field isolates from three South African provinces and global reference isolates. The phylogenetic tree was constructed based on concatenated nucleotide sequences of housekeeping genes (*sodB, secY, lipB* and *lipA* partial sequences), using MEGA7 and maximum likelihood method. Sequences from this study are shown in bold. The numbers at the nodes represent bootstrap values.

A consistent pattern of SNPs extending throughout the sequenced genes was observed for all genes. Using concatenated nucleotide sequences of housekeeping genes, *sodB, secY, lipB* and *lipA*, an MLST phylogenetic tree was constructed using only samples that successfully amplified all the four genes. The *sucA* gene was only successfully amplified in a small portion of the samples and was excluded from the MLST analysis. Phylogenetic tree topology showed three clades: clade 1 (Southern and East African and Caribbean isolates), clade 2 (West African isolates) and clade 3 (unique South African isolates: Omatjenne, Kümm2 and Riverside). It was observed that some samples were not 100% identical to any of the reference isolates (samples blocked in red in Figure 3). However, they were all found to belong to the southern and East African or worldwide clade (clade 1). No province-specific grouping was found among the analysed field isolates, suggesting that similar isolates are found all over South Africa.

## Discussion and conclusion

Heartwater, caused by *E. ruminantium*, is one of the major causes of livestock loss in sub-Saharan Africa with mortalities up to 90% (Allsopp 2015). The high genetic diversity of *E. ruminantium* is a hindrance to the development of an efficient vaccine (Cangi et al. 2016). Understanding the genotypic characteristics of *E. ruminantium* isolates currently circulating is a prerequisite to developing an efficient vaccine.

In this study, we screened adult *A. hebraeum* ticks obtained from cattle in three provinces of South Africa to characterise circulating *E. ruminantium* isolates. The overall occurrence of *E. ruminantium* in the three South African provinces is 22% which is higher than Cameroon, Benin and Mozambique (6.6%, 6% and 15%), respectively (Esemu, Ndip & Ndip 2018; Guo et al. 2018; Matos et al. 2019). Mtshali et al. (2015) reported the infection rate of *A. hebraeum* ticks collected in KZN to be slightly over 25%, which is similar to the 22% reported in the current study. Guo et al. (2019) did not detect any *E. ruminantium* from *A. hebraeum* ticks collected in Msinga Mountain View dip tank, an area that is surrounded by the three sample collection areas of our study in KZN. However, in the current study, the overall occurrence of *E. ruminantium* in sample collection sites in KZN was 22%. A possible explanation for the discrepancy in these findings might be that Guo et al. (2019) may have missed low parasitaemia positive ticks as they used conventional nested pCS20 PCR, which is less sensitive than pCS20 Sol1 qPCR (Cangi et al. 2017), which was used in our study.

Altogether, there was genetic variation in the five analysed housekeeping genes among the *E. ruminantium* field isolates. The topology of the phylogenetic tree had three *E. ruminantium* clades: clade 1 consisting of isolates from the current study – Southern and East African and Caribbean (Gardel); clade 2 with exclusively West African isolates and clade 3 with the unique South African isolates. The most conserved genes were *lipB* and *sodB* as all the analysed isolates were 100% identical to the previously known *E. ruminantium* isolates. The *sodB* gene was also highly conserved in *E. ruminantium* isolates obtained from *A. variegatum* samples in Burkina Faso (Adakal et al. 2010) and Uganda (Nakao et al. 2011), and it has been suggested that *sodB* might be a promising target for a molecular diagnostic test to identify *E. ruminantium* (Nakao et al. 2011). Adakal et al. (2010) found *lipB* to have the most polymorphic sites out of the genes used in our study; however, the current study found *lipB* to be one of the most conserved genes.

**FIGURE 4:**
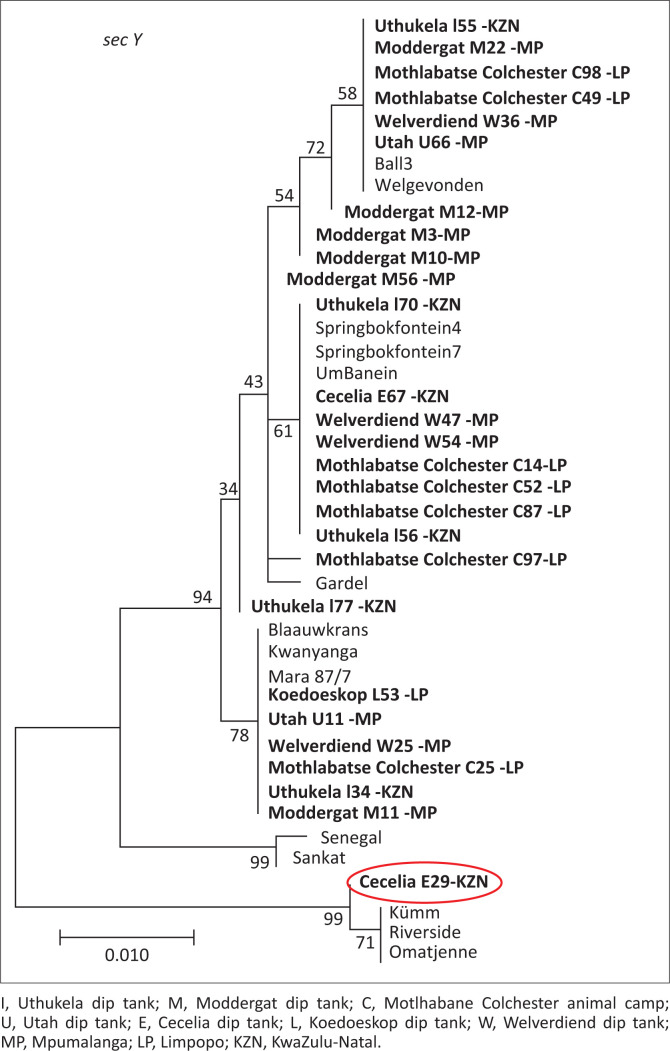
Phylogenetic tree showing relationship between *Ehrlichia ruminantium* field isolates from three South African provinces and global reference isolates based on *secY* gene. Study isolate E29 from Cecelia dip tank KwaZulu-Natal province circled in red clustered with the unique South African isolates.

Study isolate E29 from Cecelia dip tank, KZN, was observed to be 99.8% identical to *E. ruminantium* isolates, Omatjenne, Kümm2 and Riverside, and grouped with the same isolates for *secY* gene (circled red in Figure 4). The same phylogenetic tree topology of the three isolates (Omatjenne, Kümm2 and Riverside) grouping together was observed by Steyn and Pretorius (2020) and Liebenberg et al. (2020). Omatjenne genotype was isolated from a *Hyalomma truncatum* female tick collected from cattle in the heartwater-free Otjiwarongo district of Namibia and contributed to the heartwater seropositivity of cattle in the area. Initially, the isolate was apathogenic until it was a passage through three generations of *A. hebraeum* and caused a disease similar to heartwater in sheep (Du Plessis 1990). The same Omatjenne genotype was detected in blood from healthy sheep in LP, South Africa, which was seropositive for heartwater (Allsopp et al. 1997). In our study, Kümm2 clustered with Omatjenne, implying they are indeed closely related and concur with the findings of Zweygarth et al. (2002).

Kümm2 isolate is a component of the Kümm isolate and has an exact 16S rDNA genotype of Omatjenne isolate (Zweygarth et al. 2002). The isolate was obtained from naturally infected goats in Rust de Winter, an area bordering LP and northern Gauteng provinces in South Africa (Du Plessis 1981). The Riverside isolate was obtained from the blood of a sick Angora goat in one of the heartwater endemic areas in South Africa, a farm called Riverside situated in Makhanda (formerly known as Grahamstown) in the EC (Steyn & Pretorius 2020).

Omatjenne, Kümm2 and Riverside genotypes can be initiated *in vitro* in the tick cell line IDE8, but not in bovine endothelial cells (Liebenberg et al. 2020), and they lack certain open reading frames which are present in other *E. ruminantium* isolates (Allsopp et al. 2003). Liebenberg et al. (2020) found variations in the membrane protein families of Omatjenne, Kümm2 and Riverside, which may play a critical role in their ability to be propagated in other cells. Study isolate E29 could exhibit these unique genotypic characteristics because it is closely related to the three isolates. These genotypes were previously found in the EC (Steyn & Pretorius 2020) and borders of LP and northern Gauteng provinces (Du Plessis 1981) in South Africa. In our study, the unique genotype was observed in KZN. The movement of animals from one province to another, allowed by trade, may play a role in disseminating the unique genotype provincially or some isolates have evolved.

Using the MLST scheme to characterise *E. ruminantium* isolates reveals genetic diversity, SNPs throughout the sequenced regions and three main lineages. The SNPs do not change the makeup and function of the protein encoded by the genes. The three main lineages, one made up of the worldwide isolates, the other comprising of only West African isolates and the last one consisting of the unique South African isolates, remain throughout the years despite the occurrence of recombination or evolution of isolates in the field. As there are many genotypes at any location at any given time, especially in Southern Africa, there is a need for regular surveillance to understand the driving force of lack of cross-protection between *E. ruminantium* isolates. Multilocus sequence typing can clearly distinguish the South African genotypes from the distinct West African genotype, and to the best of our knowledge, this is the first report of using a panel of housekeeping genes to characterise *E. ruminantium* field isolates from ticks in three South African provinces.

## Appendix 1

**TABLE 1-A1:** Prevalence of *E. ruminantium* in *A. hebraeum* ticks collected from cattle in the study areas.

Sample collection areas	Samples processed (*n*)	pCS20 qPCR positive	
		*N*	%
**Mpumalanga**			
Utah	100	8	8
Welverdiend	150	20	13
Moddergat	58	25	43
Geoderede-S	87	22	25
**Total**	**395**	**75**	**19**
**Limpopo**			
Groblersdal	28	4	14
Koedoeskop	102	23	23
Motlhabane Colchester	136	46	34
**Total**	**266**	**73**	**27**
**KwaZulu-Natal**			
Cecelia	140	31	22
Uthukela	83	19	23
Mpungamhlophe	120	24	20
**Total**	**343**	**74**	**22**

qPCR, real-time polymerase chain reaction.
